# Correlation Between Gut Microbiota and Testosterone in Male Patients With Type 2 Diabetes Mellitus

**DOI:** 10.3389/fendo.2022.836485

**Published:** 2022-03-25

**Authors:** Shuang Liu, Ruying Cao, Luna Liu, Youyuan Lv, Xiangyu Qi, Zhongshang Yuan, Xiude Fan, Chunxiao Yu, Qingbo Guan

**Affiliations:** ^1^ Department of Endocrinology, Shandong Provincial Hospital, Cheeloo College of Medicine, Shandong University, Jinan, China; ^2^ Shandong Clinical Research Center of Diabetes and Metabolic Diseases, Shandong Key Laboratory of Endocrinology and Lipid Metabolism, Shandong Prevention and Control Engineering Laboratory of Endocrine and Metabolic Diseases, Jinan, China; ^3^ Department of Endocrinology, ChangQing People’s Hospital, Jinan, China; ^4^ Department of Biostatistics, School of Public Health, Cheeloo College of Medicine, Shandong University, Jinan, China; ^5^ Department of Endocrinology, Shandong Provincial Hospital Affiliated to Shandong First Medical University, Jinan, China

**Keywords:** T2DM, gut microbiota, dysbiosis, testosterone deficiency, male

## Abstract

**Objective:**

This study aimed at investigating the association between testosterone levels and gut microbiota in male patients with type 2 diabetes mellitus (T2DM) and providing a new strategy to elucidate the pathological mechanism of testosterone deficiency in T2DM patients.

**Methods:**

In an observational study including 46 T2DM male patients, the peripheral venous blood and fecal samples of all subjects were collected. The V3–V4 regions of bacterial 16S rDNA were amplified and sequenced. Alpha and beta diversities were calculated by QIIME software. The possible association between gut microbial community and clinical indicators was assessed using the Spearman correlation coefficient. The association between the relative abundance of bacteria and testosterone levels was discovered using linear regression analysis in R language.

**Results:**

There was no substantial difference in alpha and beta diversity. *Blautia* and *Lachnospirales* were significantly much higher in the testosterone deficiency group. Linear regression analysis showed that the abundance of *Firmicutes* at the phylum level and *Lachnospirales* at the order level were negatively correlated with testosterone level. After correcting for C-reactive protein (CRP) and homeostatic model assessment of insulin resistance (HOMA-IR), the relative abundance of *Lachnospirales* still had a significant negative correlation with testosterone level. Meanwhile, at the genus level, *Lachnoclostridium*, *Blautia*, and *Bergeyella* had a statistically significant negative association with testosterone level, respectively. *Blautia* was positively associated with FPG and total cholesterol level. *Streptococcus* was found positively associated with insulin, connecting peptide, and index of homeostatic model assessment of insulin resistance.

**Conclusion:**

T2DM patients with testosterone deficiency have different gut microbiota compositions compared with T2DM patients alone. Low serum testosterone patients tend to have an increased abundance of opportunistic pathogens, which may be related to the occurrence and development of testosterone deficiency.

## Introduction

Type 2 diabetes mellitus (T2DM) is a global epidemic that is affecting the health of the population (1). As a chronic disease, T2DM has been reported associated with several complications, one of which is hypogonadism, which has a high prevalence and may be linked to a number of comorbidities (2), such as loss of libido, erectile dysfunction, feebleness, despondency, irritability, anemia, and sleep disturbance (3). According to statistics, hypogonadism affects 20%–80.4% of males with T2DM (4), which is often manifested as testosterone deficiency. The high rate of low serum testosterone in type 2 diabetic patients has been linked to defects in the hypothalamic–pituitary–gonadal (HPG) axis in previous studies (5). 10 randomly selected individuals in a small sample of 34 hypogonadal males with diabetes received magnetic resonance imaging that revealed no abnormalities in the hypothalamus or pituitary gland (5). To date, the pathogenesis of decreased serum testosterone levels and the reasons for the individual differences in these patients with type 2 diabetes remain unclear. Therefore, it is important to find other potential risk factors which affect testosterone levels and explore the promising therapy for testosterone deficiency.

In recent years, research has found a close connection between the quantity and activity of the gut microbiota and type 2 diabetes (6). As reported, changes in the gut microbiome composition and their affiliated metabolites, such as trimethylamine N-oxide and lipopolysaccharide, lead to an imbalance of gastrointestinal homeostasis, resulting in aberrant metabolite synthesis, inflammatory state, glycometabolic disruption, and insulin resistance (7, 8). On the other hand, some studies supposed that the gut microbiota may affect the testicular function and regulate androgen production through endotoxin or its metabolites (9). Steroid-17,20-desmolase, a classic steroid-metabolizing enzyme, has been found in some bacteria, including *Clostridium *(10). *In vitro*, several bacterial strains have been demonstrated to be able to digest androgens, such as *Comamonas testosteroni*, which can feed on testosterone (11). The testosterone levels of old male mice might be restored by feeding them purified microorganisms like *Lactobacillus reuteri *(12). However, the association between gut microbiome composition and androgen deficiency in T2DM male patients is still not clarified.

Accordingly, this study is designed to compare the microbiota diversity and composition in T2DM patients according to its testosterone levels, which help us verify the relationship between specific bacteria and testosterone level in T2DM patients, to explore the promising therapy for male hypogonadism and whether the intestinal bacteria have the capacity to be a potential marker for men hypogonadism.

## Patients and Methods

### Patients

**Figure 1:**
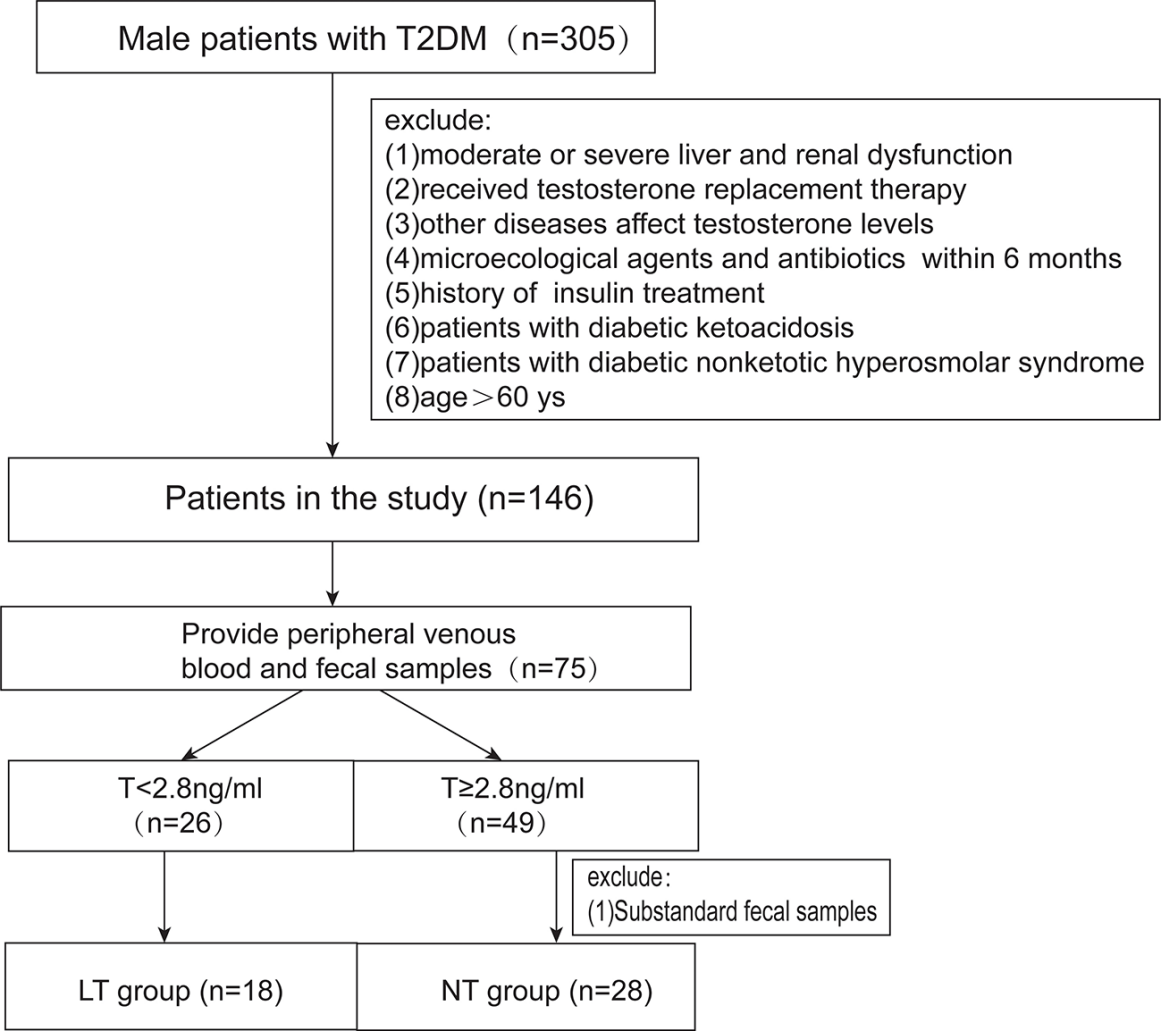
The schematic diagram of this study.

This cross-sectional study was approved by the Shandong Provincial Hospital ethical committee. All subjects signed a written inform consent. We assessed male patients with diabetes who visited Shandong Provincial Hospital between August 2018 and October 2018. The World Health Organization’s criteria were used to diagnose classic T2DM. Patients were excluded if they (1) have moderate or profound liver and renal dysfunction; (2) have a history of hypogonadotropic hypogonadism, thyroid dysfunction, ramex, testicle, and epididymis injury, abnormal karyotype, or other disease which can affect testosterone levels; (3) had undergone any antibiotic or hormone therapy in the previous 6 months; (4) have diabetic ketoacidosis or diabetic non-ketotic hyperosmolar syndrome; and (5) are >60 years old. According to the US Endocrine Society recommendation (13, 14), a low testosterone level is defined as <2.8 ng/ml. The cohort involved 46 patients. 18 patients had low testosterone levels (LT group), and 28 patients had normal testosterone levels (NT group) (**Figure 1**).

### General Information Collection

The age, duration of T2DM, medical history (hypertension, coronary heart disease), and smoking and alcohol status of the participants were all documented. Weight was recorded by adjusting 0.1 kg. Physical status was evaluated by body mass index (BMI). The blood pressure was taken after a sitting posture for 30 min. All individuals had venous blood samples drawn in the morning following an overnight fast of at least 8 h.

### Serum Parameter and Sex Hormone Analysis

The total testosterone, follicle-stimulating hormone (FSH), luteinizing hormone (LH), prolactin (PRL), insulin (INS), and connecting peptide (C-P) were tested using electrochemiluminescent procedures (Cobas E601; Roche, Basel, Switzerland). The uric acid (URIC), total cholesterol (TC), triglyceride (TG), low-density lipoprotein cholesterol (LDL-C), glycosylated hemoglobin A1c (HbA1c), fasting blood glucose (FPG), and C-reactive protein (CRP) levels were measured using enzymatic methods with an ARCHITECT ci16200 Integrated System (Abbott, Abbott Park, IL, USA). HOMA-IR = FPG(mmol/L) × INS(μIU/mL)/22.5. Testosterone secretion index (TSI) = T(nmol/L)/LH(IU/L). (The conversion criterion for testosterone is 1 ng/ml = 0.288184 nmol/l) (15).

### Clinical Data Statistical Analysis

Continuous data with a normal distribution are provided as mean values with standard deviation (SD), whereas variables without a normal distribution are shown as median values with an interquartile range (IQR; 25th–75th percentiles). The percentages are used to represent categorical variables. A t-test for continuous variables was performed for variables having a normal distribution. For variables without a normal distribution, the non-parametric Mann–Whitney U-test was used. Fisher exact tests were used to compare the constituent ratio. All results were analyzed by R software (Version 4.1.2). *p* < 0.05 was considered to be statistically significant.

### Fecal Sample Collection and Sequencing

Fecal samples collected in centrifuge tubes were stored at –20°C and then transferred to a –80°C freezer within 24 h. DNA was extracted from the fecal samples, and sequencing libraries were generated using TruSeq DNA PCR-Free Sample Preparation Kit (Illumina, San Diego, CA, USA) to amplify the V3–V4 regions (Novogene Co., Ltd., Beijing, China).

### Data Analyses of Gut Microbiota

#### Alpha Diversity

Alpha diversity was reflected by Chao1, Observed-species, Simpson, and Shannon indexes. The Chao1 index was chosen to determine Community richness, while the Simpson and Shannon indexes were utilized to determine Community diversity. All these indexes in our samples were calculated with QIIME software (Version 1.9.0) and displayed with the ggpubr package in R software (Version 4.1.2).

#### Beta Diversity

The beta diversity analysis was performed to assess how different samples differed in terms of species complexity. Beta diversity was calculated on QIIME software. Principal coordinate analysis (PCoA) was performed based on Bray–Curtis distance to get principal coordinates and visualize multidimensional data. A distance matrix of previously collected sample data was translated into a new set of orthogonal axes, with the first primary coordinate displaying the largest variance factor, the second main coordinate displaying the second maximum factor, etc. PCoA analysis was displayed by WGCNA package, stat packages, and ggplot2 package in R software.

The effect size (LEfSe) of linear discriminant analysis (LDA) was utilized to find the bacterial taxa and metabolism-related clinical factors that differed significantly across groups. Logarithmic LDA values > 2.0 were set as the threshold for differential flora identification.

#### Linear Regression Analysis

Linear regression analysis was used to analyze the relationship between testosterone and the abundance of microbiota in T2DM. Stepwise linear regression analysis was used to identify the association between testosterone levels and related risk factors. All results were analyzed by R software. *p* < 0.05 was considered to be statistically significant.

#### Model Prediction and Correlation Analysis

A comparison of the random forest model based on taxa composition and the correlation between gut microbiota and clinical manifestations (Spearman’s rank correlation coefficient) was completed using the Wekemo Bioincloud (https://www.bioincloud.tech).

## Results

### Clinical and Metabolic Variables of Participants

**Table 1** summarizes the medical history and clinical and metabolic data of the participants. The age (*p* = 0.68), BMI (*p* = 0.67), LDL-C (*p* = 0.59), TC (*p* = 0.95), TG (*p* = 0.12), URIC (*p* = 0.14), HbA1c (*p* = 0.50), FPG (*p* = 0.13), INS (*p* = 0.08), CRP (*p* = 0.29), FSH (*p* = 0.88), LH (*p* = 0.47), and PRL (*p* = 0.77) were similar between the two groups. Compared with the NT group, C-P is significantly higher in the LT group (*p* = 0.002).

**Table 1:** Clinical characteristics.

Variables	LT (n = 18)	NT (n = 28)	p-value
**Age** (yr)	42.22 ± 8.10	41.36 ± 6.85	0.68
**BMI** (kg/m^2^)	26.56 ± 5.12	25.72 ± 3.93	0.67
**SBP** (mmHg)	129.78 ± 5.07	127.36 ± 3.33	0.37
**DBP** (mmHg)	89.72 ± 3.54	84.39 ± 2.03	0.16
**Biochemical parameters**			
FSH (mIU/mL)	5.98 ± 3.19	5.83 ± 2.72	0.88
LH (mIU/mL)	5.43 ± 2.23	5.98 ± 2.82	0.47
PRL (ng/mL)	12.89 ± 9.63	13.61 ± 4.48	0.77
LDL-C (mmol/L)	3.13 ± 1.24	3.31 ± 0.79	0.59
TG (mmol/L)	2.18 (1.45, 5.07)	1.66 (1.07, 2.45)	0.12
TC (mmol/L)	5.14 ± 1.91	5.11 ± 1.13	0.95
URIC (μmol/L)	392.72 ± 111.31	348.39 ± 69.52	0.14
FPG (mmol/L)	10.52 ± 3.08	9.06 ± 3.08	0.13
HbA1c (%)	9.25 ± 1.97	9.70 ± 2.52	0.50
INS (μU/mL)	10.98 ± 6.64	7.56 ± 5.14	0.08
C-P (ng/mL)	2.76 ± 1.16	1.79 ± 0.84	0.002
CRP (mg/L)	1.83 (0.67, 3.25)	1.16 (0.79, 1.71)	0.29
**Duration of diabetes** (yr, n [%])			
0–5	13 (72.2%)	18 (64.3%)	0.75
>5	5 (27.8%)	10 (35.7%)	
**Medical history** (n [%])			
Hypertension	10 (55.6%)	6 (21.4%)	0.03
Coronary artery disease	2 (11.1%)	2 (7.1%)	0.64
Fatty liver	15 (83.3%)	20 (71.4%)	0.29
**Smoking history** (n [%])			
None	4 (22.2%)	7 (25%)	0.96
Past	3 (16.7%)	4 (14.3%)	
Current	11 (61.1%)	17 (60.7%)	
**Alcohol history** (n [%])			
None	8 (44.4%)	8 (28.6%)	0.52
Social	7 (38.9%)	15 (53.6%)	
More than twice a week	3 (16.7%)	5 (19.8%)	
**Medication history** (n [%])			
Acarbose	1 (5.6%)	3 (10.7%)	0.83
Metformin	3 (16.7%)	6 (21.4%)	
Linagliptin	3 (16.7%)	1 (3.6%)	
Acarbose+ metformin	2 (11.1%)	4 (14.3%)	
Metformin + linagliptin	1 (5.6%)	2 (7.1%)	
None	8 (44.4%)	12 (42.9%)	

BMI, body mass index; SBP, systolic blood pressure; DBP, diastolic blood pressure; CAD, coronary artery disease; FPG, fasting blood glucose; INS, insulin; HbA1c, glycosylated hemoglobin A1c; URIC, uric acid; TC, total cholesterol; LDL-C, low-density lipoprotein cholesterol; TG, triglyceride.

All data except TG were normal distribution and were analyzed by t-test, expressed as mean ± SD. TG of non-normal distribution were analyzed by non-parametric Wilcoxon test and expressed as median with interquartile range (IQR). Fisher exact tests were used to compare the constituent ratio.

### Microbiota Distribution and Diversity of Gut Microbiota

We analyzed the diversity and relative abundance of taxon in the two groups to determine the distribution of intestinal microbiota in T2D males included in this study. At the phylum level, the predominant microbiota taxa in samples from two groups were *Bacteroidetes*, *Firmicutes*, and *Proteobacteria* (**Supplemental Figure 1A**). Some bacteria genera, such as *Bacteroides*, *Lactobacillus*, *Bifidobacterium*, *Blautia*, and *Escherichia–Shigella*, dominated the gut microbiota of the two groups at the genus level.

**Figure 2:**
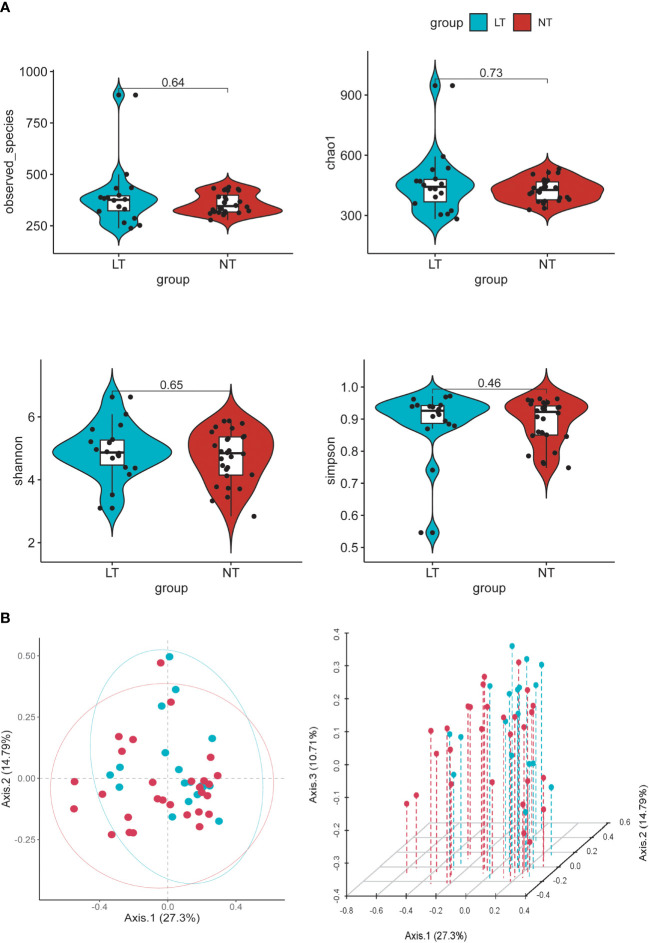
Alpha and beta diversity of the gut microbial communities from participants. **(A)** Comparison of observed OUT Numbers, chao1, shannon and simpson indexes between two groups. **(B)** Principal coordinate analysis (PCoA) of fecal microbiota, each dot represents the bacterial community composition of one individual stool sample, and the axis titles indicates the percentage variation explained (27.3% and 14.79 %, respectively).

To determine the differences in community construction and richness between the two groups, we checked the microflora diversity in the groups (using Chao1, observed species, Shannon, and Simpson indexes). However, in these indicators, there was no significant difference between the two groups (**Figure 2A**). We further compared the differences in community membership between the different groups by PCoA (**Figure 2B**). Between the NT and LT groups, there was no discernible difference in gut flora.

### Microbial Species Differed Between the LT Group and NT Group

**Figure 3:**
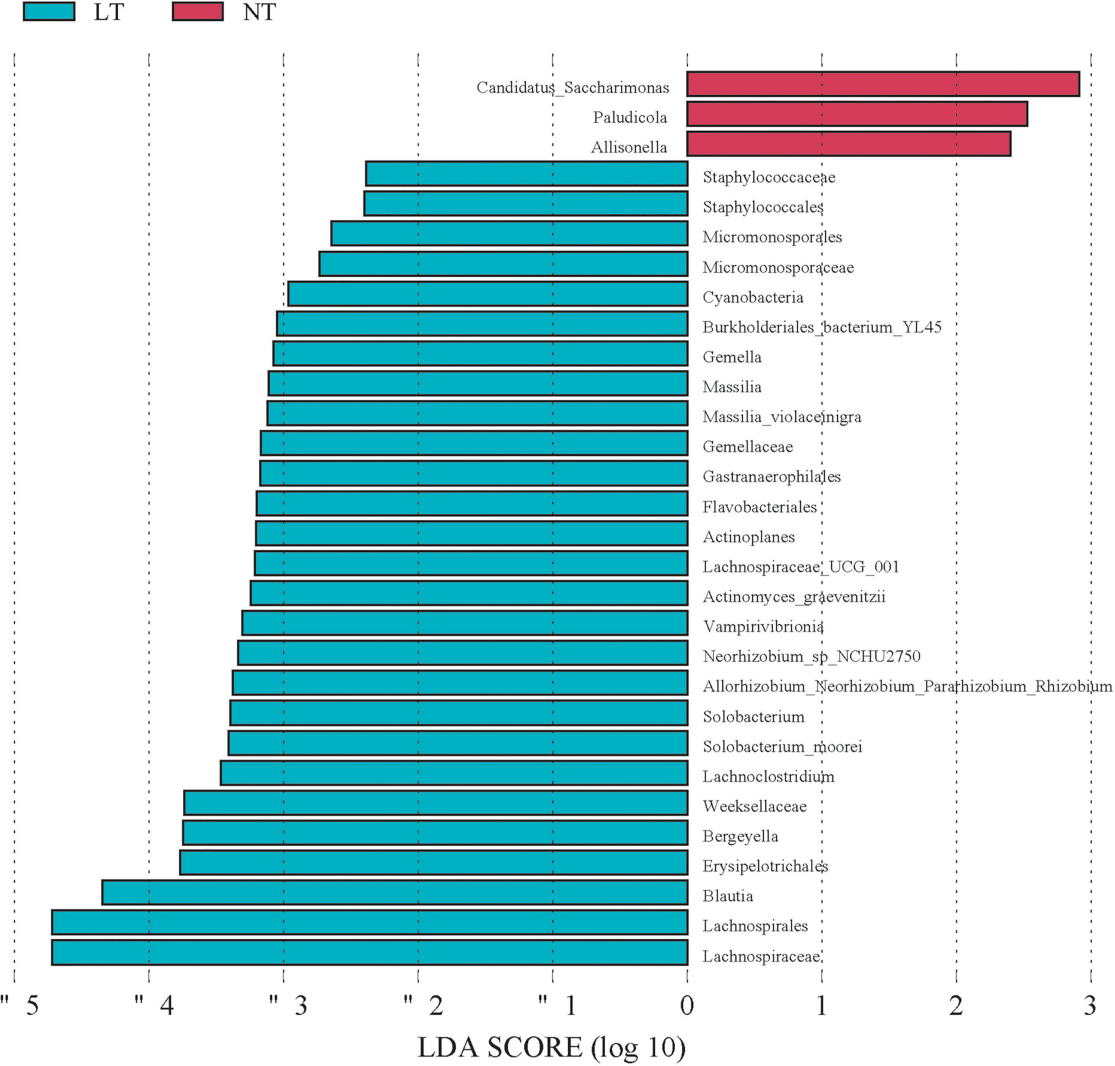
Microbial species differed between LT group and NT group. Linear discrimination analysis (LAD) effect size (LEfSE). Histogram of the LAD scores computed for differentially abundant species between the LT group and control (NT) group. The LAD scores (Log 10)>2 are listed.

We used the LEfSe method to investigate the microbe difference between the LT and NT groups. Compared with the NT group, T2D men with low testosterone had a greater abundance of *Massilia*, *Gemella*, *Lachnospiraceae_UCG_001*, *Actinoplanes*, *Allorhizobium_Neorhizobium_Pararhizobiu*, *Solobacterium*, *Lachnoclostridium*, *Parvimonas*, *Bergeyella*, and *Blautia* at the genus level, yet *Candidatus_Saccharimonas*, *Paludicola*, and *Allisonella* were the most abundant species in the NT group (**Figure 3**). These findings revealed that the LT and NT groups had statically significantly different microbial species.

### Comparison of Random Forest Model Based on Taxa Composition

**Figure 4:**
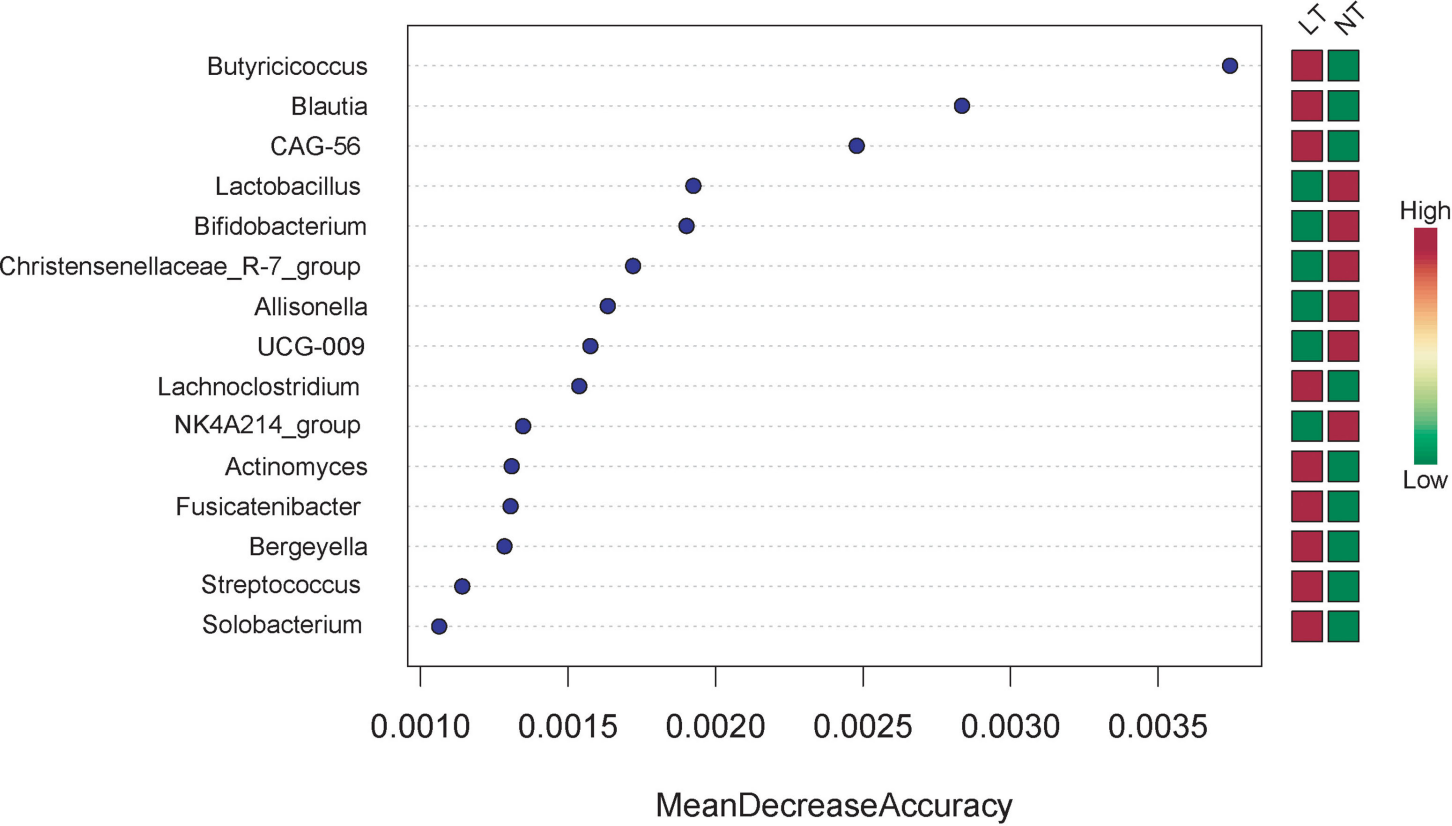
Comparison of Random Forest Model based on taxa composition.

In terms of variable importance, intestinal bacteria, including *Butyricicoccus*, *Blautia*, *CAG-56*, *Lachnoclostridium*, *Actinomyces*, *Fusicatenibacter*, *Bergeyella*, *Streptococcus*, and *Solobacterium* were the most associated with the LT group, and *Lactobacillus*, *Bifidobacterium*, *Christensenellaceae_R-7_group*, *Allisonella*, *UGC-009*, and *NK4A214_group* were the most associated with the NT group (**Figure 4**).

### The Association Between Gut Microbiota and Clinical Parameters

**Figure 5:**
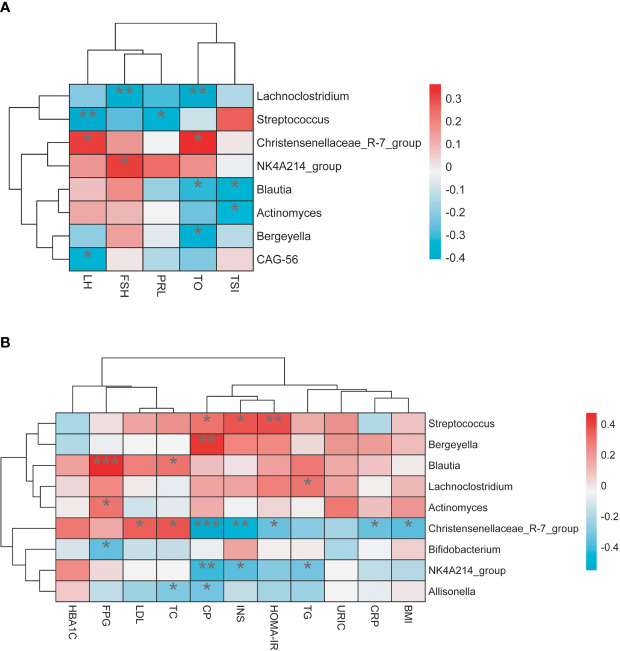
**(A)** The relationship between gut microbiota and sex hormone. **(B)** The relationship between gut microbiota and related metabolic indicators. *P < 0.05, **P < 0.01, ***P < 0.001.

We explored the relationship between these individuals’ gut microbiome and their clinical data; *Lachnoclostridium*, *Blautia*, and *Bergeyella* had a statistically significant negative association with testosterone level, respectively. Furthermore, *Lachnoclostridium is* also negatively correlated with FSH and positively correlated with TG, and *Blautia* was positively associated with fasting blood glucose (FPG) and TC level*. Streptococcus* was found to have a positive association with INS, C-P, and HOMA-IR. By contrast, the microbiota taxa related to the NT group, such as *Christensenellaceae_R-7_group*, was found positively correlated with the serum levels of testosterone and LH and negatively correlated with BMI, INS, C-P, HOMA-IR, and CRP levels. *Bifidobacterium* had a negative association with the FPG level (**Figure 5**).

### Linear Regression Analysis of Microbiota Associated With Testosterone in T2DM

**Figure 6:**
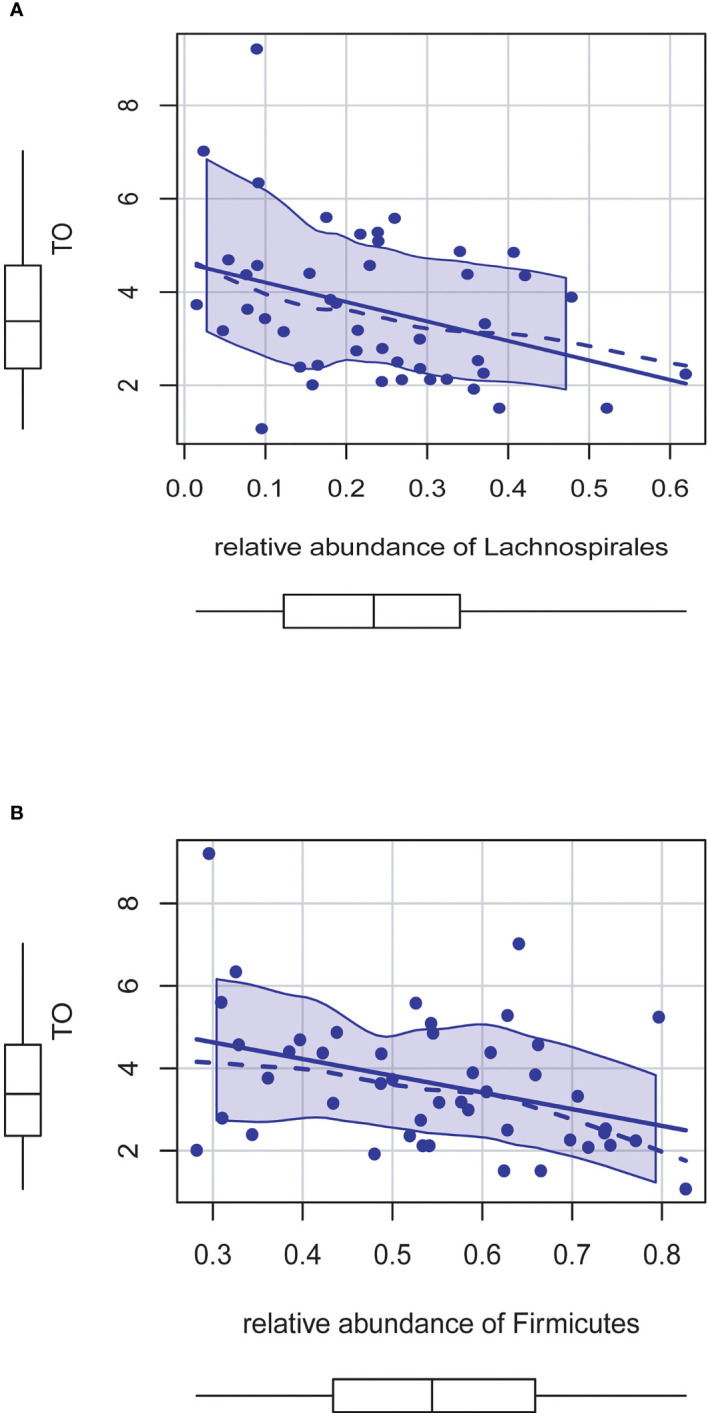
**(A)** The relative abundance of Lachnospirales is negatively correlated with testosterone levels (P = 0.015). **(B)** The relative abundance of Firmicutes is significantly negatively associated with testosterone level (P = 0.011).

Based on the above results, we found that most of the intestinal flora associated with the testosterone level group (*Blautia*, *CAG_56*, *Lachnoclostridium*, *Fusicatenibacter*) belongs to *Lachnospirales* at the order level and *Firmicutes* at the phylum level. We use linear regression analysis to assess the association between them and found that *Lachnospirales* is negatively correlated with testosterone levels (*p* = 0.015) (**Figure 6A**). We further analyzed the relationship between *Firmicutes* and testosterone level and found that at phylum levels, the relative abundance of *Firmicutes* is significantly negatively associated with testosterone level (*p* = 0.011) (**Figure 6B**).

### Stepwise Linear Regression Analysis of Risk Factors Associated With Testosterone in T2DM

The stepwise linear regression analysis was used to identify the association between testosterone levels and related risk factors. After adjustment for HOMA-IR and CRP, the relative abundance of *Lachnospirales* still had a significant negative correlation with testosterone level (**Table 2**). The relative weight model of Jeff Johnson (16) was used to evaluate the degree to which each predictive variable explained the model variance (R^2^ = 30.8%). HOMA-IR explained 34.97% of R^2^, and CRP explained 32.22% of R^2^, followed by *Lachnospirales* (32.22%) (**Supplemental Figure 2**).

**Table 2:** Stepwise linear regression analysis of risk factors associated with testosterone in T2DM.

Variables	B	SE	p
Lach	-3.267	1.584	0.045
HOMA-IR	-0.138	0.062	0.031
CRP	-0.012	0.005	0.013

Lach, relative abundance of Lachnospirales.

### The Correlation Analysis of Microbiota Taxa

**Figure 7:**
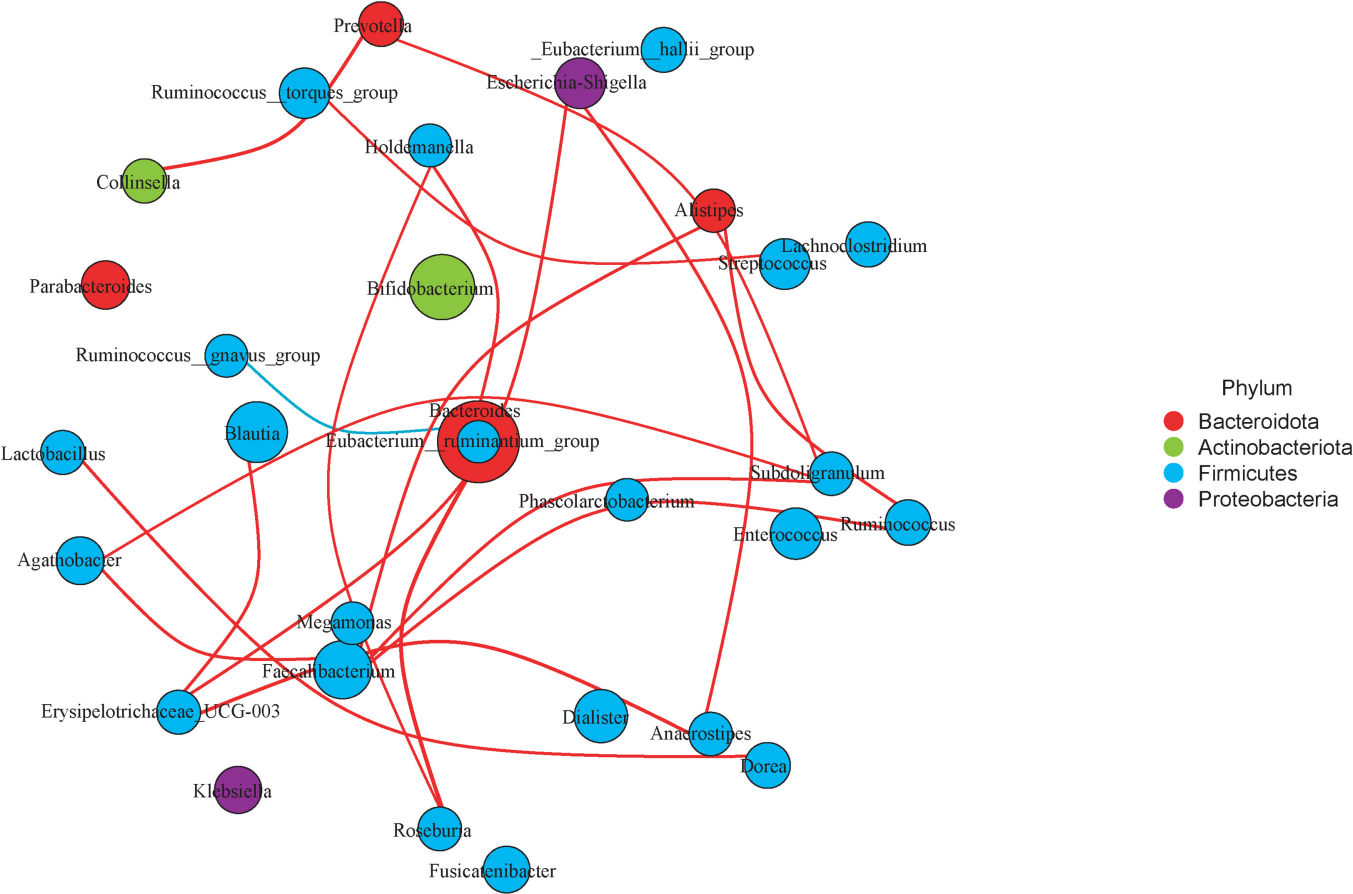
Microbiota network of LT group. Choose the top 30 microbiota taxon of LT group and detect correlations of them (Spearman’s rank correlation coefficient>0.6, adjusted P<0.05). The red lines represent a positive correlation, and the blue lines represent negative correlation.

Correlation analysis was used to decipher the relationship of microbiota taxon in the LT group. These findings were then used to build a network for detecting microbial taxon intragroup correlations (Spearman’s rank correlation coefficient > 0.6, adjusted p < 0.05). *Erysipclotrichaccae_UCG_003*, *Megamonas*, *Faecalibacterium*, *Agathobacter*, and *Roseburia* which belong to *Firmicutes* show a closely positive connection. *Ruminococcus_torques_group* had a significant positive correlation with *Streptococcus. Ruminococcus_gnavus_group* which belongs to *Firmicutes* and *Bacteroidota* shows a closely negative connection (**Figure 7**).

## Discussion

In the past 50 years, with the decline in physical activity and a shift to diets with high-fat content high-calorie foods, T2DM is increasing in prevalence (17, 18). These led to changes in metabolism and hormonal regulation (18), which lead to many complications. T2DM concomitant with testosterone deficiency may significantly affect sexual function and multiple-organ systems, leading to sarcopenia, obesity, frailty, cognitive complaints, and cardiovascular diseases (19–21). Such problems seriously undermine the mental and physical health as well as life quality of men. Subnormal testosterone levels are found inside one to two-thirds of all males with T2DM (19). The pathophysiological mechanism underpinning T2DM-induced hypogonadism has not been identified. Gut microbiota dysbiosis is a feature that is shared by both T2DM and hypogonadism (22, 23). However, the role of gut dysbiosis in testosterone deficiency with T2DM remains unclear. Our findings demonstrated that T2DM patients with testosterone insufficiency had more severe gut dysbiosis than T2DM groups, which is manifested as an increase in opportunistic pathogens and gram-negative bacteria. Furthermore, some taxa at the genus level, such as *Lachnoclostridium*, *Blautia*, and *Bergeyella*, were linked to testosterone and had strong correlations with metabolic indicators such as HOMA-IR, FPG, TC, and TG.

According to the research done in this work on the structural composition of intestinal flora, we showed that the composition of gut microbiota of testosterone deficiency patients and the NT group is different. However, there was no significant difference between the two groups in terms of α-diversity and β-diversity. At present, some studies show that the species diversity was positively associated with testosterone levels (24), but some research has not found a connection between them (25). It might be due to the limited sample size, and increasing the sample size may result in a more exact response.

LEfSe multilevel species discrimination was used to identify the important bacterial genus in the digestive tract of individuals with low testosterone levels. The microbes enriched in the NT group were critical to a stable intestinal environment. For example, *Candidatus_Saccharimonas* may help to reduce intestinal inflammation and prevent intestinal barrier dysfunction (26). By comparison, the LT group was enriched with opportunistic pathogens such as *Gemella*, *Bergeyella*, *Parvimonas*, and *Actinomyces*. According to earlier research, these infections may be linked to metabolic problems, immunological modulation, and inflammation (27, 28). Furthermore, Gram-negative bacteria such as *Massilia* and *Allorhizobium*, which were plentiful with LPS, were increased in LT patients (29, 30). LPS was associated with an increase in a number of metabolic illnesses, which are among the strongest elicitors of pro-inflammatory signaling (31). According to some studies, injecting LPS into mice reduces the synthesis and metabolism of Cyp3a11, a steroidogenesis enzyme (32); other studies showed that LPS promoted infectious orchitis (33). The various characteristics and metabolic processes of these pathogens may increase the host’s susceptibility to inflammation, resulting in testosterone deficiency. This is in line with Tremellen’s view that metabolism endotoxemia caused by gut microbiota dysbiosis might impact androgen production (29).

The impact of gut dysbiosis on testosterone levels may not be limited to inflammation. Previous research has proposed that gut microbiota may affect testosterone levels *via* the HPG axis (9). According to our results, the microbiota taxa with a higher importance in the LT group, such as *Lachnoclostridium* and *Streptococcus*, showed a stronger negative correlation with FSH and LH, respectively. It may suggest a possible mechanism that gut microbiota may affect testosterone by regulating the HPG axis. Moreover, other taxa related to the LT group, such as *Lachnoclostridium* and *Blautia*, are significantly positively correlated with HOMA-IR, TG, TC, and FPG which represent metabolic disorders, which is consistent with the results of Ren (34) and Xie (25). According to previous reports, insulin resistance and hyperlipidemia were independently associated with testosterone levels (35). We proposed that the neuroendocrine disorders caused by gut microbiota dysbiosis, together with previous findings, may indicate an underlying mechanism that explained testosterone deficiency.

We found that most of the intestinal flora associated with testosterone levels belongs to *Lachnospirales* at the order level. Further analysis shows that after adjustment for HOMA-IR and CRP, the relative abundance of *Lachnospirales* had a significant negative correlation with testosterone level. Previous research has found that the quantity of *Lachnospirales* including *Blautia* is enhanced in a variety of disorders, such as diabetes and non-alcoholic fatty liver disease. Inflammation, hyperglycemia, and obesity have all been linked to the activity of *Lachnospirales* (36–38). In germ-free (GF) mice, *Lachnospirales* were identified from the hyperglycemic obese mice, indicating its role in the development of metabolic disorders. The aforesaid species colonized GF mice, causing substantial increases in plasma glucose, as well as reduced serum insulin (36). At present, there is no research that reported that *Lachnospirales* could directly affect the synthesis or metabolism of testosterone. We suspect that the increased abundance of *Lachnospirales* may cause or represent a metabolic disorder status. Identification of *Lachnospirales* dysbiosis may be an important predictor of testosterone deficiency in T2DM male patients.

Another interesting feature of our research is that taxonomic groups of similar microorganisms like to clump together. This was in line with prior findings (39, 40). *Erysipclotrichaccae_UCG_003*, *Megamonas*, *Faecalibacterium*, *Agathobacter*, and *Roseburia* which belong to *Firmicutes* show a closely positive connection. The *Ruminococcus_torques_group* had a significant positive correlation with *Streptococcus*. These microorganisms, which have a favorable correlation in testosterone deficit individuals, may collaborate in certain pathologic situations.

This study has some limitations. Firstly, this study included participants of various ages, regions, and lifestyles, potentially resulting in heterogeneity. Secondly, because of the cross-sectional character of the study, the findings are mostly associations. Thirdly, we put much emphasis on serum testosterone to explore whether gut dysbiosis is associated with testosterone deficiency, and did not measure the contents of testosterone metabolites in the fecal samples. As a result, a well-designed randomized study investigating the clinical and metabolic consequences of hypogonadism in male T2DM patients is suggested. Despite these flaws, we believe that our research is worthwhile. Previous studies usually focused on comparing the changes of microbiota between diabetic patients and health but ignored the differences in their dietary structure. T2DM patients tend to prefer a high-fat diet. Thus, choosing T2DM patients in our study could exclude the effect of lifestyle on microbes as much as possible.

Our findings may help to better understand the effects of gut microbiota on testosterone metabolism, as well as the most effective way to prevent hypogonadism in diabetic male patients. In the future, evaluating the relative metabolites in fecal samples is helpful to explore the underlying reasons for the correlation between microbiota and testosterone level. Focusing on the exploration of potential probiotics, microbiota prescription, and microbial regulator to treat males with andrological diseases will be an appealing prospect.

## Data Availability Statement

The original contributions presented in the study are included in the article/**Supplementary Material**. Further inquiries can be directed to the corresponding authors.

## Ethics Statement

This cross-sectional study was approved by the Shandong Provincial Hospital ethical committee. Written informed consent was obtained from all participants.

## Author Contributions

Conceptualization, SL and RC. Data curation, RC. Analysis, SL and ZY. Validation and visualization, SL, LL, and XF. Writing—original draft, SL and RC. Writing—review and editing, SL, RC, YL, XQ, CY, and QG. All authors contributed to the article and approved the submitted version.

## Funding

This work was supported by grants from the National Natural Science Foundation (81770860) and the Key Research and Development Plan of Shandong Province (2017CXGC1214).

## Conflict of Interest

The authors declare that the research was conducted in the absence of any commercial or financial relationships that could be construed as a potential conflict of interest.

## Publisher’s Note

All claims expressed in this article are solely those of the authors and do not necessarily represent those of their affiliated organizations, or those of the publisher, the editors and the reviewers. Any product that may be evaluated in this article, or claim that may be made by its manufacturer, is not guaranteed or endorsed by the publisher.
