# Supplementary material for: Correlation Between Gut Microbiota and Testosterone in Male Patients With Type 2 Diabetes Mellitus
Source: Front Endocrinol (Lausanne). 2022 Mar 25;13:836485. doi: 10.3389/fendo.2022.836485 (PMC8990747; doi:10.3389/fendo.2022.836485)
Supplement: Supplementary file 1 [file DataSheet_1.pdf]

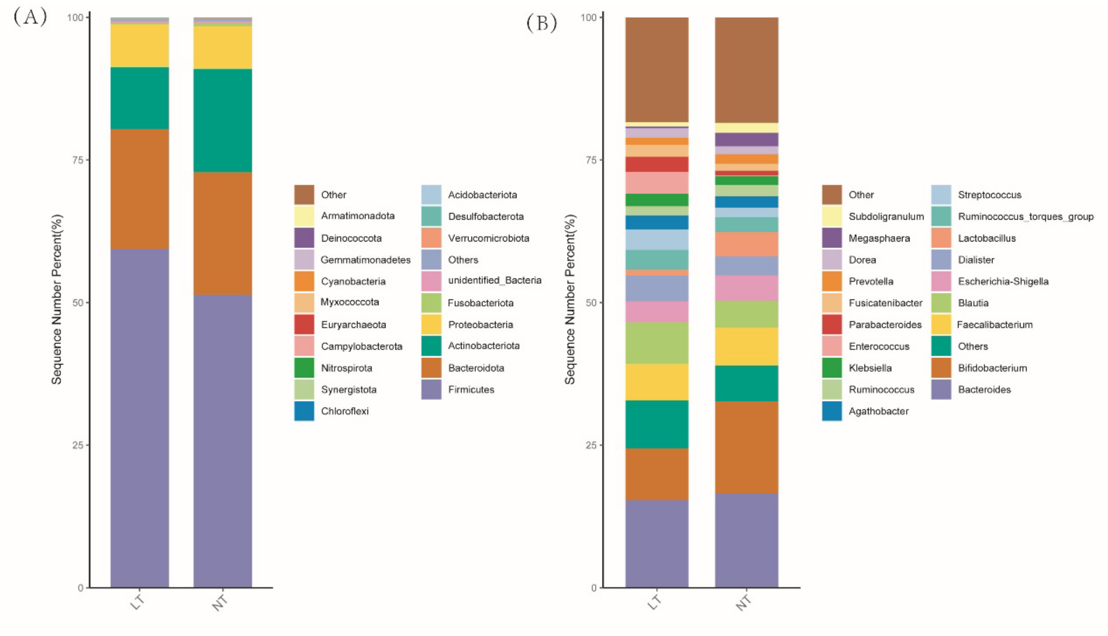

Supplemental Fig.1 Microbiota Composition in different groups

(A) The major microbiota taxa in samples of two groups at the phylum level. (B) The major microbiota taxa in samples of two groups at the genus level.

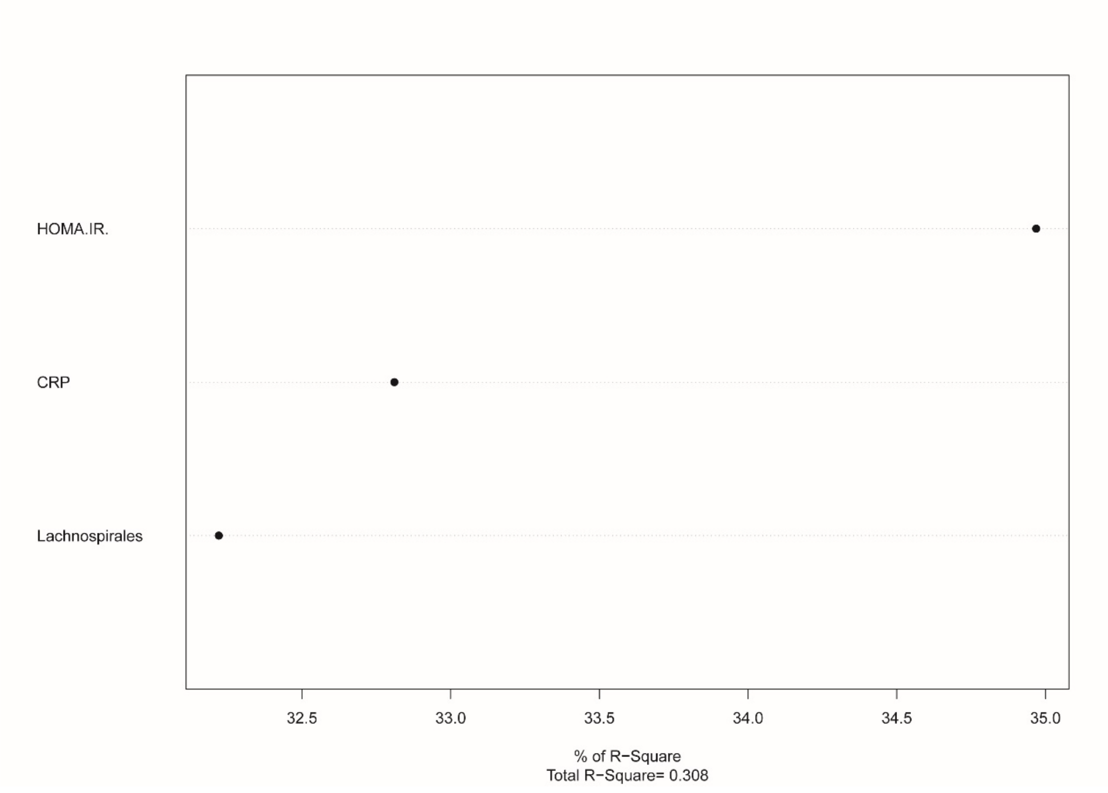

Supplemental Fig.2 Relative Importance of Predictor Variables. R^2=30.8%, HOMA-IR explained 34.97% of R2 and CRP explained 32.22% of R2, followed by Lachnospirales (32.22%)
